# Impact of telehealth during the COVID-19 pandemic on clinical and nutritional conditions of adolescents with cystic fibrosis

**DOI:** 10.36416/1806-3756/e20230397

**Published:** 2024-05-08

**Authors:** Lavínia Mayara da Silva Reis, Aline Antunes de Cerqueira Pinheiro, Maurício Antônio da Silva, Christine Pereira Gonçalves, Nelbe Nesi Santana

**Affiliations:** 1. Fundação Oswaldo Cruz - Fiocruz - Rio de Janeiro (RJ) Brasil.

## TO THE EDITOR:

Cystic fibrosis (CF) is a severe, progressive, and multisystemic genetic disease that requires a regular, in-person treatment routine.^1^ ^,^ ^2^ In 2020, with the advent of the COVID-19 pandemic, given the quarantine situation imposed by the authorities, self-care was encouraged, and services were provided remotely, thus establishing a routine of multiprofessional teleconsultations. Given this, we sought to verify how the impacts of telehealth during the COVID-19 pandemic reflected on the clinical and nutritional characteristics of adolescents monitored at a CF referral center (CFRC) in the state of Rio de Janeiro, Brazil.

A retrospective longitudinal study was carried out in which data from adolescents with CF (≥ 12 years of age) who were monitored between March and December of 2020 were evaluated. Teleconsultations were scheduled by the pulmonology team, with the participation of other specialties, such as physiotherapy, nutrition, nursing, and social assistance, all of which being involved in the treatment of CF. Teleconsultations were carried out via video calls or phone calls, depending on the availability of the patient. In addition to that, emergency consultations were available when requested by the patient or caregiver if deemed necessary. Telemonitoring was also carried out in patients considered to have more severe disease. The configuration of this service was only available in 2020.

The variables studied were as follows: BMI, height for age (H/A), percentile of arm muscle circumference (pAMC), percentile of BMI for age (pBMI/A), percentile of FEV_1_ in percentage of the predicted value (pFEV_1_%), percentile of predicted FVC (pFVC%), and FEV_1_/FVC ratio. Results of these variables were compiled from the years 2017, 2018, 2019, 2021, and 2022 in order to establish the progression of the changes of the variables studied. Adolescents whose necessary data for the research were unavailable in their medical records were excluded from the study. Continuous data were presented as means and standard deviations, as were categorical data as absolute and relative frequencies. The mean differences between the years were also calculated, and the t-test for paired samples was used to compare them. Statistical significance was set at p < 0.05. This study was submitted to and approved by the research ethics committee of the institution (CAAE no. 52272115.0.0000.5269; opinion no. 1,431,706).

We evaluated 35 adolescents with a mean age of 11.0 ± 2.7 years, 54.3% of whom were female. It is possible to notice a reduction in the mean values of H/A and BMI/A over the years, which became more pronounced in the years following the online service period (Table 1). At the same time, the percentage of malnourished people according to the pAMC increased proportionally in the same years. In relation to lung function, a decline was also observed in the mean values of pFEV_1_% and pFVC%, while the FEV_1_/FVC ratio maintained stationary values throughout the study period. When evaluating year-to-year differences, statistical significance was observed only between 2018 and 2019 and between 2021 and 2022 in the pFEV_1_% and pFVC%. There were no statistically significant differences between the period before the interruption of face-to-face meetings and after the return of consultations at the institution.

**Table 1:** Clinical and nutritional variables of adolescents with cystic fibrosis between 2017 and 2022 (N = 35).

Variable	2017	2018	p (2017-2018)	2019	p (2018-2019)	2021	p (2019-2021)	2022	p (2021-2022)
BMI	17.5 ± 3.43	18.0 ± 3.66	0.0062	18.1 ± 3.24	3.2443	19.3 ± 3.18	0.000	19.4 ± 3.48	0.7440
H/A	32.8 ± 24.99	31.1 ± 26.25	0.219	30.6 ± 27.16	0.7246	26.0 ± 24.60	0.5195	27.6 ± 25.39	0.6216
pBMI/A	45.7 ± 30.85	43.8 ± 32.58	0.3027	40.2 ± 30.47	0.1283	36.5 ± 29.41	0.4552	32.4 ± 28.06	0.2121
pAMC < 5, %	29.4	35.3		48.6		54.3		51.7	
pFEV_1_%	82.0 ± 14.89	82.0 ± 14.06	0.8183	79.0 ± 17.98	0.0395	78.0 ± 18.30	0.5519	74.0 ± 19.59	0.0385
pFVC%	92.0 ± 13.05	91.0 ± 12.33	0.5436	87.0 ± 15.0	0.0065	89.0 ± 14.78	0.9911	85.0 ± 15.54	0.0536
FEV_1_/FVC, %	83.0 ± 9.93	84.0 ± 8.08	0.3399	84.0 ± 9.03	0.4251	84.0 ± 10.86	0.5373	83.0 ± 12.94	0.7057

H/A: height for age; pBMI/A: percentile of BMI for age; pAMC: percentile of arm muscle circumference; pFEV_1_%: percentile of predicted FEV_1_; and pFVC%: percentile of predicted FVC.

Although there was a decline in nutritional and lung function variables in the years studied, there were no statistically significant differences in the same characteristics between the period before and after the interruption of in-person consultations. This fact can be attributed to the almost immediate start of teleconsultations with the CFRC team that are knowledgeable of the specificities of patients with chronic and complex diseases.

It is noted that due to the progressive nature of the disease, nutritional and lung function data of these patients had already followed a course of decline even before the pandemic period. Some studies have shown high malnourishment indices in adolescents, especially in those with CF.^3^ ^,^ ^4^ Adolescence in itself is a period of psychological and physiological changes and cognitive development, and experiencing it with a chronic and progressive disease impacts these changes even more. Given that nutritional status directly impacts functional capacity and, consequently, quality of life, adolescence requires primary attention not only from the multidisciplinary team, but also from the caregivers of these individuals.^5^ ^-^ ^7^

Because individuals with CF are considered a risk group vulnerable to complications from COVID-19, priority was given to keeping their health conditions as stable as possible through teleconsultations, telemonitoring, and encouragement of social isolation. Such strategies were extremely important to avoid worsening of lung function during that time. After the return of in-person consultations, the adolescents returned to the hospital frequently and returned to school, which explains the recurrence of the pattern of decline in the variables studied.

Costa et al.^8^ observed that most CF patients adhered to teleconsultations, demonstrating the relevance of remote assistance in the pandemic period. Teleconsultations were made available to the patients monitored quarterly, in a multidisciplinary manner, with different specialties that cover the treatment of CF, at the CFRC, where the study was carried out. This organization encouraged individuals with CF to join the online modality.

The distribution of CFRCs in Brazil is heterogeneous, the majority of them being in state capitals, so patients need to travel long distances to attend appointments.^9^ Teleconsultation and telemonitoring in CF can be considered a possibility of accessing healthcare wherever the patient is and was considered a convenient option in comparison with outpatient care.^10^

Gur et al.^11^ evaluated the perception of patients with CF and their families regarding the experience of remote care, and, although there were challenges, such as difficulty in accessing the Internet, patients were satisfied with the intervention and improved communication with the team. The authors highlighted that remote care is an acceptable and viable intervention.

Corroborating the literature, in our study, telehealth proved to be an important tool in the treatment of CF, since the values of the studied clinical and nutritional parameters were maintained. This finding can be justified by the rapid implementation of multidisciplinary teleconsultations at the CFRC, which evaluated and monitored individuals, carrying out interventions whenever necessary.
